# Interprofessional collaboration in the care of children with multiple disabilities: a cross-sectional survey of rehabilitation clinicians

**DOI:** 10.3389/fped.2026.1787352

**Published:** 2026-04-10

**Authors:** Rania Alkahtani, Nisreen Naser Al Awaji, Lama Al Bashir, Sarah Alahmari, Raghad Alwuraykah, Reem Alotaibi, Lujain Almutairi, Rawaa Alhijazi, Wajd Dhafer Alqahtani, Layan Sulaiman Algharbi

**Affiliations:** 1 Department of Health Communication Sciences, College of Health and Rehabilitation Sciences, Princess Nourah Bint Abdulrahman University, Riyadh, Saudi Arabia; 2 Department of Community Health, Faculty of Medicine, Universiti Kebangsaan Malaysia, Kuala Lumpur, Malaysia; 3 Department of Rehabilitation Sciences, College of Health and Rehabilitation Sciences, Princess Nourah bint Abdulrahman University, Riyadh, Saudi Arabia

**Keywords:** audiology, children with multiple disabilities, interprofessional collaboration, multidisciplinary practice, occupational therapy, physiotherapy, Saudi Arabia, speech-Language pathology

## Abstract

**Purpose:**

Children with multiple disabilities (CMD) present complex, overlapping needs that place exceptional demands on interprofessional rehabilitation systems. Although interprofessional collaboration is widely endorsed, its implementation remains inconsistent, with particular challenges in the integration of audiology services. This study explored clinicians’ perceptions, behaviors, and barriers toward multidisciplinary practice (MDP), with a focus on differences between professional groups.

**Methods:**

A cross-sectional online survey was administered to licensed physiotherapists, occupational therapists, speech-language pathologists, and audiologists practicing in pediatric rehabilitation services in Saudi Arabia. The questionnaire assessed demographics, role familiarity, MDP implementation, perceived benefits and barriers, and training needs. Descriptive statistics were generated, and chi-square tests were used to examine associations between profession and key variables.

**Results:**

A total of 284 professionals participated. Significant differences were observed between professions in role familiarity, with audiologists reporting the lowest levels. While most respondents reported engaging in collaboration, audiologists were the least likely to participate in multidisciplinary teams. Key barriers included limited awareness of professional roles, staff shortages, and inadequate training, with variation across disciplines.

**Conclusion:**

Interprofessional collaboration is valued but inconsistently implemented in pediatric rehabilitation for CMD, with notable disparities in the integration of different professions, particularly audiology. Strengthening practice requires clarifying professional roles, providing structured interprofessional training, and fostering supportive organizational cultures. These findings underscore the need for systemic interventions to enable truly comprehensive and integrated care for children with multiple disabilities.

## 1 Introduction

Children with multiple disabilities (CMD) present with two or more co-occurring impairments across physical, sensory (e.g., hearing), communication, cognitive, or developmental domains, often experiencing complex and overlapping functional challenges (1, 2). For the purpose of this study, CMD were operationally defined as complex clinical presentations involving two or more co-occurring impairments across physical, sensory (e.g., hearing), communication, cognitive, or developmental domains, requiring input from two or more rehabilitation disciplines. This definition encompasses a spectrum of presentations, from strictly co-occurring physical and sensory impairments to broader definitions including complex behavioral or cognitive needs, as different definitions can influence perceived barriers and necessary team composition. The World Health Organization's International Classification of Functioning, Disability and Health (ICF) framework emphasizes that effective rehabilitation should not only target impairments but also promote activity, participation, and interaction with environmental and personal factors (3–5). Achieving such holistic outcomes requires contributions from multiple rehabilitation disciplines, such as physiotherapy, occupational therapy, speech-language pathology, and audiology, working collaboratively rather than in isolation (6, 7).

Interprofessional models of care are increasingly recognized as essential for children with complex needs (7–9). When teams coordinate goal setting, information sharing, and care plans, outcomes such as functional gains, continuity of care, and family satisfaction tend to improve (10, 11). In pediatric rehabilitation, evidence suggests that interprofessional collaboration enhances communication between professionals, reduces duplication of efforts, and allows for the earlier identification of emerging impairments that might otherwise be overlooked (7). However, despite broad endorsement, the real-world implementation of multidisciplinary practice remains uneven across different settings and professional groups.

Across healthcare systems, pediatric rehabilitation research has tended to focus on specific diagnostic groups, with comparatively less attention to the interprofessional processes required to support children with multiple disabilities. Within Saudi Arabia, existing studies reflect a similar pattern, emphasizing condition-specific services while identifying gaps in service integration and limited role awareness across rehabilitation professions (3, 12–17). These observations align with broader international concerns regarding the need to move from fragmented, profession-dominant models toward more integrated, team-based approaches to care (18, 19)

Accordingly, this study examines rehabilitation clinicians' perceptions, behaviors, and perceived barriers to interprofessional collaboration in the care of children with multiple disabilities. By comparing perspectives across physiotherapy, occupational therapy, speech-language pathology, and audiology, the study aims to identify profession-specific disparities and system-level factors that shape multidisciplinary practice.

## 2 Material and methods

### 2.1 Study design and setting

This study employed a cross-sectional, questionnaire-based design to assess perceptions of roles and interprofessional collaboration among rehabilitation professionals caring for children with multiple disabilities in Saudi Arabia. Data were collected between May 2025 and November 2025. The survey targeted the four core pediatric rehabilitation disciplines: physiotherapy, occupational therapy, speech-language pathology, and audiology.

### 2.2 Participants and eligibility

A total of 284 rehabilitation professionals participated in this study. Inclusion criteria were: (i) licensed/credentialed physiotherapists, occupational therapists, speech-language pathologists, or audiologists; (ii) active clinical practice in Saudi Arabia (public hospital, private hospital/clinic, or specialized rehabilitation center); and (iii) age ≥18 years. Exclusion criteria were students, non-clinical staff, and duplicate submissions (screened via timestamps and response-pattern checks). Respondents who indicated that they had not previously seen children with multiple disabilities were allowed to complete only the applicable sections via branching logic.

For the purposes of the survey, clinicians were asked to reflect on their experience with children who present with multiple co-occurring disabilities requiring input from more than one rehabilitation discipline. No restriction was placed on specific diagnostic labels; instead, CMD was operationalized as complex clinical presentations involving overlapping motor, sensory, communication, cognitive, or developmental needs typically managed through multidisciplinary rehabilitation services.

### 2.3 Recruitment and procedure

A non-probability convenience sample was recruited through professional networks and clinician-focused social media channels (e.g., WhatsApp, X/Twitter). The survey specifically targeted licensed rehabilitation professionals, physiotherapists, occupational therapists, speech-language pathologists, and audiologists, who were actively practicing in pediatric rehabilitation settings in Saudi Arabia, including public hospitals, private hospitals/clinics, and specialized rehabilitation centers, and who had clinical experience with children with multiple disabilities. The invitation described study aims, eligibility, voluntary participation, and an estimated completion time of approximately 10 min, and included a link to the anonymous survey. After reviewing the information sheet on the first page, participants provided informed consent electronically and proceeded to the questionnaire. No incentives were offered. As the survey platform used did not record incomplete or partial responses, only fully completed submissions were captured, a formal response rate could not be calculated. This limits the ability to assess the proportion of individuals who began but did not complete the survey.

The number of invitations sent to each professional group was not tracked separately, as the survey was disseminated broadly without discipline-specific targeting or quota allocation. The resulting unequal group sizes in the final sample, physiotherapists (*n* = 117, 41.2%), audiologists (*n* = 66, 23.2%), occupational therapists (*n* = 62, 21.8%), and speech-language pathologists (*n* = 39, 13.7%), are likely to reflect the relative proportions of these disciplines within the Saudi pediatric rehabilitation workforce and the differential reach of the distribution channels used, rather than deliberate differential recruitment.

### 2.4 Instrument (questionnaire)

The questionnaire reflected the final survey shared by the investigators and comprised the following sections:

1. Demographics (Q1-Q7): age, gender, occupation, professional classification, nationality, workplace, and years of professional experience.
2. Perceptions and role familiarity (Q8-Q12): Likert-type items assessing familiarity with each profession’s role and agreement with the importance of multidisciplinary practice. Items Q8-Q11 included a “Not applicable, I am a [discipline]” option to prevent self-rating.
3. Multidisciplinary practice-current implementation (Q13-Q22; “Multidisciplinary I”): presence and frequency of collaboration; collaborating specialties (multi-select); confidence and authority to refer; team communication rating; perceived effectiveness of multidisciplinary practice; perceived benefits, challenges, and resources (multi-select with open-ended options).
4. Multidisciplinary practice-barriers among non-collaborators (Q23-Q24; “Multidisciplinary II”): factors hindering engagement (multi-select) and an open-ended prompt on perceived advantages.
5. Training and future perspectives (Q25-Q30): prior education/training in multidisciplinary practice; belief that multidisciplinary practice training is needed; recommended training topics (open-ended); enablers to future collaboration (multi-select); willingness to recommend initiatives; and satisfaction with the current status of multidisciplinary practice.

Branching/skip logic: Q13 routed respondents who reported no collaboration (or no exposure) to “Multidisciplinary II” (Q23-Q24), whereas collaborators proceeded to Q14-Q22.

### 2.5 Content validity and clarity

The draft instrument underwent expert peer review by clinicians from the four core pediatric disciplines (physiotherapy, occupational therapy, speech-language pathology, and audiology) to ensure clarity, content coverage (role familiarity, collaboration behaviors, barriers/facilitators, training), and face validity. The most extensive input was provided by a speech-language pathologist with 11 years of clinical experience and an audiologist with approximately 20 years of clinical experience in pediatric rehabilitation. Minor wording and ordering edits were incorporated following feedback. Response formats included single-choice Likert scales, categorical items, multi-select checklists, and open-ended questions.

### 2.6 Data management

Survey data were collected anonymously and exported to IBM SPSS Statistics (version 25) for cleaning and analysis. Multi-select responses (e.g., barriers to implementation, resources needed for collaboration, and factors hindering engagement) were dummy coded as separate binary variables (1 = selected; 0 = not selected) and analyzed quantitatively; the resulting frequencies are reported in the tables. “Not applicable” responses on role familiarity items were treated as missing for analyses of that construct. The questionnaire also included several free-text open-ended items (e.g., perceived advantages of multidisciplinary practice, recommended training topics, and additional barriers or resources not captured by the checklists). These responses were reviewed and analyzed using a content analysis approach, in which two researchers independently examined the responses, identified recurring themes, and resolved discrepancies by consensus. Representative quotations and thematic summaries derived from this process are integrated into the results narrative where relevant.

#### 2.6.1 Missing data handling

Descriptive statistics used available-case denominators. Inferential analyses applied pairwise deletion to maximize the use of observed data while preserving the sample size across tests.

### 2.7 Outcomes and variable coding

Pre-specified, practice-oriented dichotomizations were used to support clear interpretation and adequate cell counts for inferential analyses. Dichotomization of selected Likert-scale variables was undertaken to enhance interpretability and to meet distributional requirements for chi-square testing across professional subgroups. This approach aligns with the study's exploratory aim of identifying broad patterns of support and perceived effectiveness rather than modeling fine-grained ordinal differences. The rationale for this approach, as requested by reviewers, is that adjacent Likert categories were collapsed to facilitate decision-making (e.g., distinguishing between supportive vs. not supportive responses) and to meet the distributional requirements for contingency table analyses, particularly when examining associations between professional group and key outcomes.

The following dichotomizations were applied:

- Importance of multidisciplinary practice (Q12): “Strongly agree/Agree” vs. other (Neutral/Disagree/Strongly disagree).
- Necessity of referral (Q16): “Strongly agree/Agree” vs. other.
- Perceived effectiveness of multidisciplinary practice (Q19): “Effective/Very effective” vs. other (Unsure/Slightly/Not effective).
- Satisfaction with current multidisciplinary practice (Q30): “Satisfying/Very satisfying” vs. other.
- Willingness to recommend initiatives (Q29): “Very/Moderately likely” vs. other.
- Role familiarity (Q8–Q11): “Very/Moderately familiar” vs. “Somewhat/Not familiar” (excluding “Not applicable”).
- Collaboration frequency (Q14): “Always/Frequently” vs. “Sometimes/Rarely/Never.”

Factors associated with outcomes included age (categorized), gender, occupation (physiotherapist/occupational therapist/speech-language pathologist/audiologist), professional classification (specialist/senior specialist/consultant/other), nationality (Saudi/non-Saudi), workplace (public/private/specialized center), and years of experience (categorized).

### 2.8 Statistical analysis

All analyses were performed in SPSS version 25. Categorical variables were summarized as frequency (n) and percentage (%), and numerical variables as mean and standard deviation (SD) with ranges where informative. Associations between sociodemographic characteristics and binary outcomes were evaluated with the Pearson Chi-square test of independence. When more than 20% of cells had expected counts <5, the Monte Carlo method was used to estimate exact *p*-values. Statistical significance was set at *p* ≤ 0.05 (two-sided). Associations between collaboration frequency and selected perceptions/intentions (e.g., perceived effectiveness of multidisciplinary practice; willingness to recommend initiatives) were examined using the same approach. Cramer's V was calculated for statistically significant associations to estimate effect size.

## 3 Results

### 3.1 Participant characteristics

A total of 284 rehabilitation professionals completed the questionnaire. The demographic characteristics of the study population are presented in Table 1.

**Table 1:** Demographic characteristics by professional group (*N* = 284).

Variable	Category	Physiotherapist (*n* = 117)	Occupational Therapist (*n* = 62)	Speech-Language Pathologist (*n* = 39)	Audiologist (*n* = 66)	*χ* ^2^	df	*p*-value
Age group	Under 26	16 (13.8%)	23 (37.1%)	15 (38.5%)	5 (7.6%)	29.88	6	<.001
	27–37 years	69 (59.5%)	32 (51.6%)	17 (43.6%)	44 (66.7%)			
	38 or older	31 (26.7%)	7 (11.3%)	7 (17.9%)	17 (25.8%)			
Gender	Female	56 (47.9%)	32 (51.6%)	31 (79.5%)	45 (68.2%)	16.14	3	.001
	Male	61 (52.1%)	30 (48.4%)	8 (20.5%)	21 (31.8%)			
Professional classification	Specialist	96 (82.1%)	51 (82.3%)	22 (56.4%)	51 (77.3%)	35.18	12	<.001
	Senior specialist	14 (12.0%)	5 (8.1%)	7 (17.9%)	12 (18.2%)			
	Consultant	6 (5.1%)	0 (0.0%)	3 (7.7%)	3 (4.5%)			
	Intern/Student	1 (0.9%)	4 (6.5%)	6 (15.4%)	0 (0.0%)			
Workplace	Public hospital	79 (67.5%)	31 (50.0%)	20 (51.3%)	43 (65.2%)	8.20	6	.224
	Private hospital/Clinic	21 (17.9%)	15 (24.2%)	10 (25.6%)	14 (21.2%)			
	Specialized center	17 (14.5%)	16 (25.8%)	9 (23.1%)	9 (13.6%)			
Years of experience	Less than 3 years	15 (14.4%)	17 (32.1%)	7 (21.2%)	3 (4.8%)	30.23	9	<.001
	4–5 years	40 (38.5%)	25 (47.2%)	9 (27.3%)	24 (38.1%)			
	6–8 years	21 (20.2%)	10 (18.9%)	7 (21.2%)	18 (28.6%)			
	9 or more years	28 (26.9%)	1 (1.9%)	10 (30.3%)	18 (28.6%)			

PT, physiotherapist; OT, occupational therapist; SLP, speech-language pathologist; AUD, audiologist.

Values are *n* (%). χ^2^ = Pearson chi-square statistic. Bold *p*-values indicate statistical significance (*p* ≤ .05).

The mean age was 33.0 years (SD 6.6; range 19–53; *n* = 281), with 52.5% aged 27–37 years. Females comprised 57.7% of respondents. By discipline, physiotherapists were most represented (41.2%), followed by audiologists (23.2%), occupational therapists (21.8%), and speech-language pathologists (13.7%). Most were specialists (77.5%), Saudi nationals (95.1%), and employed in public hospitals (60.9%). Mean professional experience was 7.0 years (SD 5.5; range 1–30; *n* = 245).

To support interpretation of the findings, the distribution of key demographic variables across professional groups was examined (Table 1). Statistically significant differences were observed across professions in age [*χ*^2^(6) = 29.88, *p* < .001], gender [*χ*^2^(3) = 16.14, *p* = .001], professional classification [*χ*^2^(24) = 47.80, *p* = .003], and years of experience [*χ*^2^(9) = 30.23, *p* < .001], while workplace distribution did not differ significantly across groups [*χ*^2^(6) = 8.20, *p* = .224]. Regarding age, audiologists were the most experienced group in terms of age, with 66.7% aged 27–37 years and 25.8% aged over 38, whereas occupational therapists (37.1%) and speech-language pathologists (38.5%) had the highest proportions of participants under 26 years. In terms of gender, speech-language pathologists were predominantly female (79.5%), while physiotherapists were predominantly male (52.1%). Regarding professional experience, audiologists had the lowest proportion of early-career clinicians (less than 3 years: 4.8%) and a relatively high proportion of those with 4–5 years of experience (38.1%), whereas occupational therapists had the highest proportion of early-career clinicians (32.1%). Physiotherapists and audiologists had the highest proportions of clinicians with 9 or more years of experience (26.9% and 28.6%, respectively). Workplace distribution was broadly similar across professions, with the majority in all groups employed in public hospitals (50.0- 67.5%), though occupational therapists and speech-language pathologists had a slightly higher representation in specialized rehabilitation centers (25.8% and 23.1%, respectively) compared to physiotherapists and audiologists (14.5% and 13.6%). These demographic differences across professional groups should be considered when interpreting the findings, as they may partially account for observed profession-level variations in collaboration behaviors and perceptions.

### 3.2 Familiarity with professional roles

Familiarity with the roles of other disciplines varied considerably. The distribution of familiarity levels across different professional roles is presented in Table 2.

**Table 2:** Familiarity with other disciplines’ roles, stratified by respondent's professional group.

Role Being Rated	Respondent Profession	n	Very Familiar n (%)	Moderately Familiar n (%)	Slightly Familiar n (%)	Not Familiar n (%)	χ^2^	df	*p*-value
Physiotherapist role	Physiotherapist^a^	29	13 (44.8%)	10 (34.5%)	5 (17.2%)	1 (3.4%)	15.02	9	.090
	Occupational Therapist	58	16 (27.6%)	22 (37.9%)	15 (25.9%)	5 (8.6%)			
	Speech-Language Pathologist	38	8 (21.1%)	14 (36.8%)	10 (26.3%)	6 (15.8%)			
	Audiologist ^b^	62	9 (14.5%)	20 (32.3%)	24 (38.7%)	9 (14.5%)			
Occupational Therapist role	Physiotherapist	115	23 (20.0%)	38 (33.0%)	44 (38.3%)	10 (8.7%)	22.39	9	.008
	Occupational Therapist ^a^	24	8 (33.3%)	11 (45.8%)	4 (16.7%)	1 (4.2%)			
	Speech-Language Pathologist	38	5 (13.2%)	16 (42.1%)	14 (36.8%)	3 (7.9%)			
	Audiologist ^b^	64	5 (7.8%)	15 (23.4%)	32 (50.0%)	12 (18.8%)			
Speech-Language Pathologist role	Physiotherapist	116	22 (19.0%)	33 (28.4%)	36 (31.0%)	25 (21.6%)	23.41	9	.005
	Occupational Therapist	62	7 (11.3%)	23 (37.1%)	25 (40.3%)	7 (11.3%)			
	Speech-Language Path. ^a^	9	6 (66.7%)	1 (11.1%)	0 (0.0%)	2 (22.2%)			
	Audiologist	66	19 (28.8%)	16 (24.2%)	20 (30.3%)	11 (16.7%)			
Audiologist role	Physiotherapist	114	19 (16.7%)	28 (24.6%)	37 (32.5%)	30 (26.3%)	23.11	9	.006
	Occupational Therapist	61	6 (9.8%)	19 (31.1%)	23 (37.7%)	13 (21.3%)			
	Speech-Language Pathologist	37	13 (35.1%)	11 (29.7%)	7 (18.9%)	6 (16.2%)			
	Audiologist ^a^	12	6 (50.0%)	1 (8.3%)	5 (41.7%)	0 (0.0%)			

Self-ratings were excluded via “Not applicable, I am a [profession]” branching logic. *n* = number of respondents providing a valid familiarity rating for that role. χ^2^ = Pearson chi-square statistic for the association between respondent profession and familiarity level across all four groups. Bold *p*-values indicate statistical significance (*p* ≤ .05).

^a^ Interpret with caution: *n* < 30, as most respondents in this group correctly selected “Not applicable” for their own profession.

^b^ A small number of audiologists (*n* = 4 for PT role; *n* = 2 for OT role) incorrectly selected a “Not applicable” option for another profession; these responses were excluded from analysis.

“Very familiar” responses were highest for physiotherapists (24.6%), followed by speech-language pathologists (21.3%), audiologists (19.7%), and occupational therapists (17.0%). “Not familiar” was most frequent for audiologists (21.9%), indicating that audiology was the least understood discipline. Notably, 21.9% of participants were not familiar at all with the audiologist's role, compared to 17.8%, 11.2%, and 10.8% who were not familiar with the roles of speech-language pathologists, physiotherapists, and occupational therapists, respectively.

Cross-tabulation of familiarity by respondent profession revealed more nuanced patterns. Regarding familiarity with the physiotherapist role, audiologists reported the lowest familiarity, with 38.7% only slightly familiar and 14.5% not familiar at all, compared to physiotherapists themselves (44.8% very familiar) and occupational therapists (27.6% very familiar); this difference was not statistically significant [*χ*^2^(9) = 15.02, *p* = .090]. Familiarity with the occupational therapist role differed significantly across groups [*χ*^2^(9) = 22.39, *p* = .008], with audiologists again reporting the lowest familiarity (50.0% slightly familiar; 18.8% not familiar) compared to occupational therapists (33.3% very familiar) and physiotherapists (20.0% very familiar). Familiarity with the speech-language pathologist role also differed significantly [*χ*^2^(9) = 23.41, *p* = .005]: physiotherapists had the highest proportion of “not familiar” responses (21.6%), while speech-language pathologists themselves reported high familiarity (66.7% very familiar) and audiologists showed moderate familiarity (28.8% very familiar). Finally, familiarity with the audiologist role differed significantly across groups [*χ*^2^(9) = 23.11, *p* = .006]: physiotherapists had the highest proportion of “not familiar” responses (26.3%), followed by occupational therapists (21.3%), while speech-language pathologists (35.1% very familiar) and audiologists themselves (50.0% very familiar) reported higher familiarity levels.

Taken together, these stratified findings indicate that audiologists consistently reported lower familiarity with the roles of other professions, and that the audiologist role was itself the least understood by physiotherapists and occupational therapists, a pattern that has important implications for referral practices and team communication.

### 3.3 Interprofessional collaboration and perceived importance

While a vast majority of respondents across all groups agreed on the importance of a multidisciplinary approach, the practice of collaboration varied significantly. Tables 3, 4, 5 summarize the key aspects of interprofessional collaboration.

**Table 3:** Perceived importance of multidisciplinary practice, stratified by professional group (Q14).

Professional Group	n	Strongly Agree n (%)	Agree n (%)	Neutral n (%)	Disagree n (%)	Strongly Disagree n (%)	χ^2^	df	*p*-value
Physiotherapist	117	32 (27.4%)	32 (27.4%)	25 (21.4%)	19 (16.2%)	9 (7.7%)	31.21	12	.002
Occupational Therapist	62	20 (32.3%)	22 (35.5%)	4 (6.5%)	11 (17.7%)	5 (8.1%)			
Speech-Language Pathologist	39	22 (56.4%)	6 (15.4%)	4 (10.3%)	2 (5.1%)	5 (12.8%)			
Audiologist	66	18 (27.3%)	29 (43.9%)	12 (18.2%)	5 (7.6%)	2 (3.0%)			

χ^2^ = Pearson chi-square statistic for the association between professional group and response category. Percentages may not sum to 100% due to rounding.

**Table 4:** Working in collaboration with other specialties, stratified by professional group (Q15).

Professional Group	n	Yes n (%)	No n (%)	Never Seen Children with MD n (%)	χ^2^	df	*p*-value
Physiotherapist	117	55 (47.0%)	37 (31.6%)	25 (21.4%)	12.82	6	.046
Occupational Therapist	62	43 (69.4%)	12 (19.4%)	7 (11.3%)			
Speech-Language Pathologist	39	27 (69.2%)	9 (23.1%)	3 (7.7%)			
Audiologist	66	33 (50.0%)	20 (30.3%)	13 (19.7%)			

MD, multiple disabilities.

χ^2^ = Pearson chi-square statistic. Percentages may not sum to 100% due to rounding.

**Table 5:** Frequency of collaboration with other disciplines, stratified by professional group (Q16).

Professional Group	n	Always n (%)	Frequently n (%)	Sometimes n (%)	Rarely n (%)	Never n (%)	χ^2^	df	*p*-value
Physiotherapist	117	46 (39.3%)	20 (17.1%)	29 (24.8%)	11 (9.4%)	9 (7.7%)	27.50	15	.025
Occupational Therapist	62	14 (22.6%)	22 (35.5%)	17 (27.4%)	5 (8.1%)	1 (1.6%)			
Speech-Language Pathologist	39	9 (23.1%)	15 (38.5%)	7 (17.9%)	5 (12.8%)	2 (5.1%)			
Audiologist	66	18 (27.3%)	9 (13.6%)	23 (34.8%)	8 (12.1%)	7 (10.6%)			

χ^2^ = Pearson chi-square statistic. Percentages may not sum to 100% due to rounding. A small number of respondents (*n* = 7) selected no response and are excluded.

Regarding the perceived importance of MDP (Q14), agreement levels differed significantly across professional groups [*χ*^2^(12) = 31.21, *p* = .002]. Speech-language pathologists expressed the strongest endorsement, with 56.4% strongly agreeing and 15.4% agreeing (combined: 71.8%). Occupational therapists also showed strong agreement (strongly agree: 32.3%; agree: 35.5%; combined: 67.8%), followed by audiologists (strongly agree: 27.3%; agree: 43.9%; combined: 71.2%). Physiotherapists showed the most variability, with 27.4% strongly agreeing, 27.4% agreeing, but 21.4% neutral and 16.2% disagreeing.

Regarding whether respondents worked in collaboration with other specialties (Q15), a statistically significant association was found between professional group and collaborative practice [*χ*^2^(6) = 12.82, *p* = .046]. Occupational therapists (69.4%) and speech-language pathologists (69.2%) were the most likely to report working collaboratively, followed by audiologists (50.0%) and physiotherapists (47.0%). Notably, physiotherapists had the highest proportion of respondents who had never seen children with multiple disabilities in their clinic (21.4%), compared to audiologists (19.7%), occupational therapists (11.3%), and speech-language pathologists (7.7%).

The frequency of collaboration (Q16) also differed significantly across groups [*χ*^2^(15) = 27.50, *p* = .025]. Physiotherapists had the highest proportion reporting “always” collaborating (39.3%), while occupational therapists (35.5%) and speech-language pathologists (38.5%) more frequently reported collaborating “frequently.” Audiologists were the most likely to report “sometimes” collaborating (34.8%) and had the highest proportion of “never” collaborating (10.6%) among those who had seen children with multiple disabilities.

### 3.4 Barriers to and effectiveness of multidisciplinary practice

Tables 6, 7, 8 present a consolidated view of the barriers to, and the perceived effectiveness of, multidisciplinary practice.

**Table 6:** Team communication and merits of multidisciplinary practice.

Category	Item	Percent (%)	*p*-value
Team Communication Rating	Excellent	27.8	0.111
	Good	51.9	
	Fair	16.5	
	Poor	3.8	
Merits of the Multidisciplinary Approach	Coordinated treatment goals	71.5	>0.05
	Improved communication	64.9	
	Holistic understanding of needs	55.0	

*p*-values reflect chi-square tests of association between professional group and each outcome. No statistically significant differences were observed across professional groups for merits items (*p* > 0.05).

**Table 7:** Barriers to multidisciplinary practice.

Barrier category	Item	Percent (%)
Challenges to Implementation	Lack of awareness about other professions’ duties	48.7
	Staff shortages	48.7
	Limited training	42.3
Resources Needed for Collaboration	Clear role guidelines	63.2
	Joint training opportunities	54.6
	Standardized communication protocols	49.3
Factors Hindering Engagement (Non-collaborators)	Staff shortage	55.1
	Limited resources	46.2
	Limited training	42.3

**Table 8:** Perceived effectiveness and satisfaction.

Category	Item	Percent (%)	*p*-value
Perceived Effectiveness	Very effective	28.9	>0.05
	Effective	28.9	
	Unsure	19.5	
Satisfaction with Current Status	Satisfying/Very satisfying	22.5	>0.05
	Unsatisfying/Very unsatisfying	42.3	
	Neutral	35.2	

No statistically significant differences were observed across professional groups for perceived effectiveness or satisfaction (*p* > 0.05).

Most participants (71.5%) agreed that the primary merit of the multidisciplinary approach was that it allows for coordinated treatment goals across disciplines, followed by improved communication and collaboration among providers (64.9%), and a more holistic understanding of patient needs (55.0%). Additional benefits were described by participants in their own words, including:

“It allows for a comprehensive and holistic treatment plan that addresses all aspects of a child's disabilities, including physical, cognitive, emotional, and social needs. This approach ensures that all aspects of the child's development are considered and coordinated by a team of experts working together towards common goals.”

“The multidisciplinary team fosters collaboration and communication among professionals, leading to coordinated care and consistent support for the child and their family.”

“The multidisciplinary approach can offer emotional and psychological support for both the child and their family. Dealing with multiple disabilities can be challenging and overwhelming, but having a team of professionals working together can provide reassurance, guidance, and encouragement throughout the treatment process.”

Across all disciplines, the most commonly cited barriers to implementing multidisciplinary practice were a limited awareness of other professions' roles (48.7%), staff shortages (48.7%), and inadequate training (42.3%). Additional barriers identified through open-ended responses included lack of management support, high caseloads with insufficient staffing, limited time for interprofessional discussion, and lack of leadership interest in improving practice quality. To overcome these challenges, participants identified several key resources: clear guidelines for the roles and responsibilities of each team member (63.2%), joint training opportunities for different disciplines (54.6%), standardized communication protocols (49.3%), and shared electronic medical records (30.3%).

Regarding authority to refer, 87.9% of participants reported having the authority to refer children with multiple disabilities to other specialties, while 12.1% did not. Those without referral authority indicated that referrals were governed by physician orders and Ministry of Health approval, with specialists limited to documenting recommendations for consultants to action.

For those who did not engage in any collaboration, the main factors hindering engagement were staff shortage (55.1%), limited resources (46.2%), and limited training (42.3%). Additional open-ended responses noted that not all cases were perceived to require a multidisciplinary approach, and that some clinicians worked in single-discipline settings (e.g., audiology-only or physiotherapy-only clinics).

While there was broad agreement on the effectiveness of the multidisciplinary approach and overall satisfaction levels (*p* > 0.05), the rating of team communication did not significantly differ between groups (*p* = 0.111), with 79.7% rating it as “good” or “excellent”.

### 3.5 Training and education in multidisciplinary practice

Table 9 summarizes the findings related to training and education in multidisciplinary practice.

**Table 9:** Training and education in multidisciplinary practice.

Training Metric	Category	Percent (%)
Prior Specific Training or Education	Yes	60.6
	No	39.4
Belief that Therapists Should Receive MDP Training	Strongly agree/Agree	55.6
	Neutral	18.3
	Disagree/Strongly disagree	26.1

Prior training/education in multidisciplinary practice was reported by 60.6% of respondents. More than half (55.6%) agreed or strongly agreed that therapists working with children with multiple disabilities should receive such training, while 26.1% disagreed or strongly disagreed. Regarding the content of such training, participants identified several key topics through open-ended responses, including: communication skills between different rehabilitation disciplines, the importance and advantages of multidisciplinary treatment, the role of each specialty in assessment and management, how to develop a holistic rehabilitation program, collaboration strategies, assessment and treatment planning as a team, and goal-sharing and collaborative counseling approaches.

Regarding future collaboration, the most commonly cited factors that would encourage participants to engage in multidisciplinary practice were clearly defining the roles and responsibilities of each team member (69.0%), offering training opportunities (63.7%), involving families as active participants in multidisciplinary team meetings (51.1%), and establishing interdisciplinary teams (47.5%). Furthermore, 73.6% of participants indicated they were very or moderately likely to recommend specific initiatives to enhance multidisciplinary in their workplace.

## 4 Discussion

This study provides a comprehensive analysis of interprofessional collaboration among rehabilitation clinicians involved in the care of CMD. The findings reveal a landscape of strong professional endorsement for MDP, which is significantly undermined by inconsistencies in its implementation. While a majority of professionals reported engaging in collaboration, the practice was not consistently applied, while MDP is widely recognized as best practice (20), its integration into everyday pediatric rehabilitation remains uneven. For children with CMD, whose complex, co-occurring conditions demand a highly integrated approach, this gap between endorsement and practice is particularly concerning and suggests that the full benefits of collaborative care are not being realized (21)

### 4.1 Professional disparities and role familiarity

A primary finding is the significant disparity in professional involvement and role familiarity, with the most pronounced gap for audiology. Our analysis revealed that audiologists were the least understood by their peers and were involved in significantly fewer collaborations compared to physiotherapists and occupational therapists. This represents more than a procedural gap; for children with CMD, it constitutes a critical vulnerability in the care pathway. Many children with complex motor disabilities have a higher prevalence of co-occurring hearing impairments that can be masked by their other challenges. The under-integration of audiology, as our findings show, means these hearing impairments may go unidentified, directly limiting the effectiveness of speech-language therapy, special education, and even motor interventions that rely on a child's ability to process auditory cues (22, 23)This suggests the current model of care for CMD in the region remains disproportionately motor-focused, and addressing this imbalance requires a shift toward an interdisciplinary “total-child” approach that recognizes sensory, communication, and motor needs as interconnected (24). Notably, this finding contrasts with data from the ASHA Interprofessional Practice Survey (2021) (25), which reported that 85% of audiologists in the United States were satisfied with their degree of collaboration, a stark difference that may reflect the more structured integration of audiology within North American pediatric rehabilitation pathways, and the comparatively limited role of audiology in team-based care in the present context.

### 4.2 Systemic barriers and the impact on CMD care

One of the most nuanced findings is the paradox between clinicians' high self-rating of communication skills and their simultaneous reporting of “limited communication channels” as a major barrier. This suggests that while individual clinicians may be competent communicators, the organizational infrastructure to support systematic, scheduled interprofessional communication is lacking. For the CMD population, this is not a minor inconvenience. The absence of formal systems can lead to fragmented care plans, conflicting advice for families, and a significant increase in caregiver burden as parents are forced to become the primary integrators of care. The resources respondents identified as most helpful, clear role guidelines, joint training, and standardized communication protocols, are the foundational elements of a functional MDT. The prominence of role-unawareness as a barrier, coupled with a lack of prior MDP training, reinforces that the challenges are systemic and educational, not a reflection of individual professional failure (19). This finding is consistent with El-Awaisi et al. (26, 27), who reported that lack of awareness of other professionals’ roles and scope of practice was among the most frequently cited barriers to interprofessional collaboration among healthcare professionals in primary care settings in Qatar, suggesting that role-unawareness is a systemic challenge inherent to professional siloing in health education, rather than a context-specific phenomenon. The cross-sectional study by Jabbar et al. (28) on attitudes and barriers to interprofessional collaboration in Pakistani hospitals similarly identified staff shortages and limited training as key barriers, findings that align closely with the present study and suggest that resource constraints are a shared challenge across healthcare contexts in the region. Importantly, open-ended responses also identified leadership and management as critical barriers, with participants noting a lack of management support, disinterest from leaders in improving practice quality, and high caseloads that leave no protected time for interprofessional discussion. These findings underscore that sustainable collaboration cannot be achieved through clinician effort alone; it requires active organizational commitment and leadership investment in creating the structural conditions for teamwork.

Furthermore, a subset of participants noted that they worked in single-discipline settings, such as audiology-only or physiotherapy-only clinics, where collaboration is structurally impossible regardless of individual willingness. This highlights that barriers to MDP are not uniform across the workforce, and that service configuration and workplace design are upstream determinants that must be addressed at a policy and planning level.

### 4.3 The role of experience and need for CMD-specific competencies

The finding that clinicians with greater professional experience reported higher satisfaction with MDP suggests that, over time, they develop the informal networks needed to navigate a fragmented system. However, a well-functioning healthcare system should not rely on individual heroics or informal relationships to deliver best-practice care. The strong prioritization of joint training opportunities by the cohort indicates that early- and mid-career clinicians recognize the need for formalized structures and interprofessional education (IPE) to engage effectively. This pattern mirrors findings by Schwab-Farrell et al. (29), who found that speech-language pathologists, physiotherapists, and occupational therapists in North American settings similarly valued interprofessional collaboration but identified structural and organizational barriers, including time constraints, unclear role boundaries, and limited formal communication systems, as the primary obstacles to its realization, reinforcing that the gap between endorsement and implementation is not unique to the Saudi Arabian context but reflects a broader international challenge in interprofessional healthcare education. For the CMD population, this training must go beyond general IPE to include CMD-specific competencies, such as understanding the interplay between motor, sensory, and cognitive development, and how interventions in one domain can impact progress in another (30).

### 4.4 Implications for practice and policy: an implementation-focused approach

CMD care represents a stress test for interprofessional systems, in which fragmentation carries amplified consequences for children and their families. To move from recommendation to sustainable practice change, the implications can be framed using an implementation science lens, such as the Consolidated Framework for Implementation Research (CFIR) (31, 32).

1. Intervention Characteristics: The perceived “relative advantage” of MDP is high among clinicians, but its “complexity” is also a major barrier. To address this, organizations should focus on simplifying collaboration through standardized referral pathways and communication templates.
2. Outer Setting: The findings highlight system-level levers relevant to healthcare systems seeking to operationalize integrated, family-centered care for children with multiple disabilities, including the alignment of financial and organizational incentives to support collaborative practice.
3. Inner Setting: The most significant barriers identified in this study fall within the inner setting. There is a clear need to improve networks and communication (e.g., protected time for meetings), foster a culture of collaboration, and provide strong leadership engagement. Implementing a designated care coordinator or case manager for each family could be a powerful strategy to improve the “structural characteristics” of the care environment (33).
4. Characteristics of Individuals: The knowledge gap regarding audiology’s role highlights the need to address clinicians’ knowledge and beliefs about other professions. Targeted IPE that uses case-based learning with CMD-specific examples can improve both knowledge and self-efficacy for collaboration.
5. Process: A structured implementation process should be adopted, starting with engaging key stakeholders (including families) to co-design new workflows. This should be followed by executing the plan with dedicated resources and reflecting and evaluating its impact on both process measures (e.g., collaboration frequency) and, eventually, patient and family outcomes.

Furthermore, a deeper commitment to family-centered care is required. This imperative is reinforced by the qualitative findings of the current study, in which participants explicitly described MDP as offering emotional and psychological support to families navigating the complexity of multiple disabilities, and noted that families feel more confident and reassured when a coordinated team is working toward shared goals. These participant perspectives align with evidence that family-centered, interprofessional care not only improves clinical outcomes but also reduces caregiver stress and enhances the family's capacity to support their child's rehabilitation at home. Adopting frameworks like the ICF “F-words” is a starting point, but it must be operationalized through structures that empower families and make them true partners in the decision-making process (34, 35)

Although the data were generated within a single healthcare system, the identified implementation determinants, particularly those related to communication structures, leadership engagement, and role clarity, align with challenges consistently reported across interdisciplinary rehabilitation settings internationally.

### 4.5 Strengths and limitations

This study has several strengths, including its inclusion of all four core pediatric rehabilitation disciplines and its examination of not only perceptions but also self-reported behaviors, barriers, and resource needs. However, several limitations should be acknowledged. First, the cross-sectional design allows only a snapshot of clinicians' perceptions and behaviors at a single point in time, which precludes the establishment of cause-and-effect relationships between the variables examined. Consequently, while associations between professional group, collaboration behaviors, and perceived barriers can be described, no directional or causal conclusions can be drawn. Longitudinal or interventional study designs would be needed to determine whether changes in, for example, training or organizational structures lead to improvements in collaboration outcomes.

Second, the unequal representation of professional groups in the sample, with physiotherapists comprising 41.2% of respondents and speech-language pathologists only 13.7%, is a recognized limitation. Although this likely reflects the proportional distribution of these disciplines within the Saudi pediatric rehabilitation workforce, the smaller sample sizes for certain groups, particularly speech-language pathologists (*n* = 39), may limit the statistical power of profession-specific comparisons and the generalizability of findings for these groups. Future studies should consider purposive or stratified sampling strategies to ensure more balanced representation across disciplines. Third, a formal response rate could not be determined, as the survey platform used did not record incomplete or partial responses; only fully completed submissions were captured. This limits the ability to assess the proportion of individuals who began but did not complete the survey, and consequently, the representativeness of the final sample. Fourth, the self-reported data may be subject to social desirability bias.

A key limitation is the focus on clinician perceptions rather than objective outcomes. Future research should investigate the direct impact of MDT on child and family outcomes, such as functional participation, quality of life, and caregiver burden or empowerment, which are critical metrics for evaluating the effectiveness of care for CMD. Additional research could also explore the impact of structured onboarding or mentorship programs for early-career clinicians and how different referral models influence service accessibility (35)

## 5 Conclusion

Interprofessional collaboration is valued but inconsistently implemented in pediatric rehabilitation for CMD in Saudi Arabia. The evidence demonstrates that these inconsistencies are driven by systemic organizational and educational barriers. For the CMD population, who require the highest level of integrated care, these gaps can lead to fragmented services, missed diagnoses, and increased family burden. To move toward truly comprehensive, participation-focused care, priorities must include implementing structured interprofessional training with CMD-specific competencies, clarifying professional roles, and developing a robust communication infrastructure, potentially including designated care coordinators. These findings highlight the urgent need for systemic interventions to ensure all children with multiple disabilities receive the integrated and family-centered care they require.

## Data Availability

The raw data supporting the conclusions of this article will be made available by the authors, without undue reservation.
